# Therapeutic Role of Tamoxifen for Triple-Negative Breast Cancer: Leveraging the Interaction Between ERβ and Mutant p53

**DOI:** 10.1093/oncolo/oyac281

**Published:** 2023-02-11

**Authors:** Lauren Scarpetti, Chetan C Oturkar, Dejan Juric, Maria Shellock, Giuliana Malvarosa, Kathryn Post, Steven Isakoff, Nancy Wang, Brian Nahed, Kevin Oh, Gokul M Das, Aditya Bardia

**Affiliations:** Massachusetts General Hospital Cancer Center, Harvard Medical School, Boston, MA, USA; Roswell Park Comprehensive Cancer Center, Buffalo, NY, USA; Massachusetts General Hospital Cancer Center, Harvard Medical School, Boston, MA, USA; Massachusetts General Hospital Cancer Center, Harvard Medical School, Boston, MA, USA; Massachusetts General Hospital Cancer Center, Harvard Medical School, Boston, MA, USA; Massachusetts General Hospital Cancer Center, Harvard Medical School, Boston, MA, USA; Massachusetts General Hospital Cancer Center, Harvard Medical School, Boston, MA, USA; Massachusetts General Hospital Cancer Center, Harvard Medical School, Boston, MA, USA; Massachusetts General Hospital Cancer Center, Harvard Medical School, Boston, MA, USA; Massachusetts General Hospital Cancer Center, Harvard Medical School, Boston, MA, USA; Roswell Park Comprehensive Cancer Center, Buffalo, NY, USA; Massachusetts General Hospital Cancer Center, Harvard Medical School, Boston, MA, USA

**Keywords:** triple-negative breast cancer, tamoxifen, estrogen receptor β, mutant p53, brain metastasis, proximity ligation assay (PLA)

## Abstract

The absence of effective therapeutic targets and aggressive nature of triple-negative breast cancer (TNBC) renders this disease subset difficult to treat. Although estrogen receptor beta (ERβ) is expressed in TNBC, studies on its functional role have yielded inconsistent results. However, recently, our preclinical studies, along with other observations, have shown the potential therapeutic utility of ERβ in the context of mutant p53 expression. The current case study examines the efficacy of the selective estrogen receptor modulator tamoxifen in p53-mutant TNBC with brain metastases. Significant increase in ERβ protein expression and anti-proliferative interaction between mutant p53 and ERβ were observed after cessation of tamoxifen therapy, with significant regression of brain metastases. This case study provides supporting evidence for the use of tamoxifen in p53-mutant, ERβ+TNBC, especially in the setting of brain metastasis.

## Teaser

Although ERβ is expressed in triple-negative breast cancer (TNBC), its functional role is not well understood. This case report examines the efficacy of the selective estrogen receptor modulator tamoxifen in p53-mutant TNBC with brain metastases.

**Key Points**

- This case report demonstrates a potential therapeutic role of tamoxifen in a molecularly defined subset of p53-mutant, estrogen receptor beta-positive triple-negative breast cancer.
- Given the limited treatment options currently available for triple-negative breast cancer, the novel findings may significantly impact the management of this breast cancer population.
- It is hoped that these results will prompt further clinical investigation into the use of tamoxifen in p53-mutant, estrogen receptor beta-positive triple-negative breast cancer to strengthen the presented findings.

## Background

Triple-negative breast cancer (TNBC) refers to tumors that lack expression of estrogen receptor alpha (ERα), progesterone receptor (PR), and absence of HER2 amplification. Clinically, patients with TNBC have aggressive tumor biology, higher risk of recurrence, and poor prognosis. Given the absence of effective therapeutic targets, chemotherapy is the mainstay of management of TNBC but is associated with significant toxicity and low response rate, particularly in the metastatic setting.

While TNBC does not express ERα, expression of estrogen receptor beta (ERβ) in 25%-30% of TNBC offers a promising opportunity for targeted antitumorigenic therapy.^1,2^ Expression of ERβ has been identified as an independent predictor of recurrence, metastasis, and mortality, and is generally regarded as a favorable clinical feature in breast cancer. ERβ1 expression, in particular, is associated with a favorable prognosis in TNBC, with reduced recurrence and improved survival outcomes.^1,3^ Importantly, tamoxifen, FDA-approved medication for ERα-positive breast cancer, is also a ligand for ERβ.^4^ It has been suggested that ERβ is anti-proliferative in nature, mainly based on exogenous overexpression of ERβ cDNA in cancer cell lines.^5–9^ However, observations in other preclinical studies indicate that ERβ instead associated with proliferation in mammary epithelial cells,^10^ breast cancer cells,^11^ and TNBC.^2^ Such inconsistent data on pro- versus anti-tumorigenic role of ERβ suggested that this protein may have bi-faceted functions.^12,13^ Using si/shRNA-mediated depletion of endogenous ERβ in breast cancer cell models, we have shown that p53 status is an important determinant of the pro- versus anti-tumorigenic duality of ERβ in breast cancer.^14^

An important characteristic of TNBC is the high prevalence (~80%) of mutations in p53.^15–17^ Preclinical studies in breast cancer cells have shown that ERβ is capable of opposing certain pro-tumorigenic functions of mutant p53.^5,9,14,18^ ERβ directly binds and antagonizes p53,^5,14,18^ and importantly, wild-type versus mutant p53 status was shown to be a determinant of the pro- versus anti-tumorigenic functional duality of ERβ.^14^ Our retrospective analysis of METABRIC (Molecular Taxonomy of Breast Cancer International Consortium) data showed that combined expression of mutant p53 and high levels of ERβ in basal-like TNBC was prognostic of better breast cancer-specific patient survival.^14^ Mutant p53 has been found to bind and inactivate tumor suppressor p73 when unrestricted.^19–24^ ERβ was shown to bind and sequester mutant p53, resulting in the reactivation of p73. Reactivated p73 is free to promote the expression of anti-tumorigenic genes, including *CDKN1A* (*p21*) and *BBC3* (*PUMA*), resulting in cell-cycle arrest and apoptosis.^14^ Notably, we showed that tamoxifen increased ERβ-mutant p53 interaction, and p73-mutant p53 interaction was reciprocally decreased leading to the reactivation of p73 as a tumor suppressor. This finding suggested that tamoxifen’s ability to inhibit the oncogenic function of mutant p53 via ERβ leading to an active p73 could be exploited for a therapeutic strategy against TNBC expressing ERβ and mutant p53. However, there has been no clinical data in support of this novel mechanism of tamoxifen function in TNBC.

Here we report an example of the efficacy of tamoxifen in TNBC. After discovery of mutant p53 variants in a metastatic nodule in October 2019, the patient’s care team hypothesized that she would benefit from tamoxifen therapy based on preclinical evidence. The patient was treated with tamoxifen for approximately 2 months, after which significant anti-proliferative mutant p53-ERβ interaction and a decrease in tumor burden were observed. This case contributes to the growing evidence supporting the use of tamoxifen in cases of TNBC expressing high levels of ERβ) along with mutant p53.

## Patient Story

The patient is a 59-year-old G3P2 woman diagnosed with primary breast cancer and later metastasis to the brain (>10 lesions), chest wall, and neck and chest lymph nodes. Pertinent medical history includes hypertension, vitamin D deficiency, degenerative joint disease, and obesity. The patient underwent genetic testing 3 times without identification of a deleterious germline mutation in *BRCA1/2*, *TP53*, or *PTEN*, and had no family history of breast or ovarian malignancy.

The first malignancy was identified in July 1991 (30 years at the age of first diagnosis) when a mass was palpated in the right breast. The patient underwent a lumpectomy with axillary lymph node dissection which revealed a stage 1b, 1-cm T1bN0 grade 3, ER—(<1%)/HER2—infiltrating ductal carcinoma (IDC) with features of medullary carcinoma. She then received adjuvant cyclophosphamide, methotrexate, and fluorouracil (CMF) for 6 cycles followed by adjuvant radiation and 2 years of tamoxifen. In September 2005, a lesion in the right breast was noted on routine mammogram. Core biopsy revealed a grade 3, triple-negative (ER–/ HER2–) IDC. The patient then underwent bilateral mastectomies, during which a 0.9-cm tumor was resected from the right breast. Final pathology revealed a stage T1bNX IDC, with associated grade 3 ductal carcinoma in situ. No malignancy was identified in either the left breast, bilateral sentinel node biopsy (SNB), or the resected lymph nodes. Four cycles of adriamycin and cyclophosphamide (AC) were completed (October—December 2005), which induced menopause.

**Figure 1.**
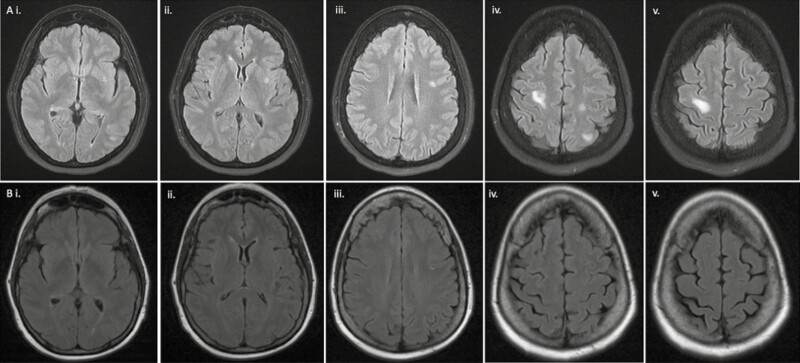
Comparison of T2 fluid-attenuated inversion recovery (FLAIR) MRI of brain metastases before (**A**) and after (**B**) tamoxifen therapy. (**A**): T2 FLAIR MRI on 10/25/2019, pre-tamoxifen therapy (i–v). (**B**): T2 FLAIR MRI on 2/10/2020, post-tamoxifen therapy (i–v).

The patient’s breast malignancies were stable until the fall of 2019 when she noted right submandibular swelling over a 5- to 7-week period. An MRI of the brain revealed multiple (>20) supratentorial and infratentorial brain metastases as well as intense uptake in submandibular, right hilar, subcarinal, right paratracheal, and anterior chest wall subcutaneous nodes, as outlined in Fig. 1A.

## Molecular Tumor Board

A core biopsy of the right neck nodule demonstrated poorly differentiated, ER–/ PR–/ HER2 2 + (FISH 0.9) carcinoma similar to the second breast primary. Average number of HER2 copies per cell was 2.1, with HER2/cep17 ratio of 0.9. Additional specimen testing revealed GATA3+, GCDFP15–, mammaglobin–, and PDL1 + (5%) expression. PDL1 expression was evaluated via immunohistochemical staining, with determination of percent tumor area comprised of PDL1-expressing immune cells. Molecular diagnostics found mutated *TP53* (i) a single nucleotide missense mutation resulting in Cys238Tyr change in the protein sequence and (ii) 18 bp/6 amino acids deletion between Pro177 and Cys182 both in the brain tumor and in the chest wall tumor tissue. Importantly, both these alterations are in the DNA-binding domain (DBD) of p53. Biopsy of the right cerebellar lesion demonstrated consistent immunohistochemistry (ER–/PR–/ HER2 2 + (FISH 0.9) and similar genomic alterations in *TP53*. No copy number variants (CNVs) of known clinical significance were detected. HER2/cep17 ratio was identical to that of the neck nodule sample, with 2.0 HER2 copies per cell on average.

The patient received 2 cycles of capecitabine but had disease progression in the brain. She then received whole-brain radiotherapy. Tamoxifen was added to the radiation therapy given preclinical data demonstrating efficacy. Repeat imaging demonstrated a decrease in size and conspicuity of multiple brain lesions, as well as chest wall subcutaneous nodes (Fig. 1B). A repeat biopsy was obtained which confirmed poorly differentiated, ER–/ PR–/ HER2 negative carcinoma, *TP53* mutations, as well as evidence of pharmacodynamic effect with tamoxifen.

**Figure 2.**
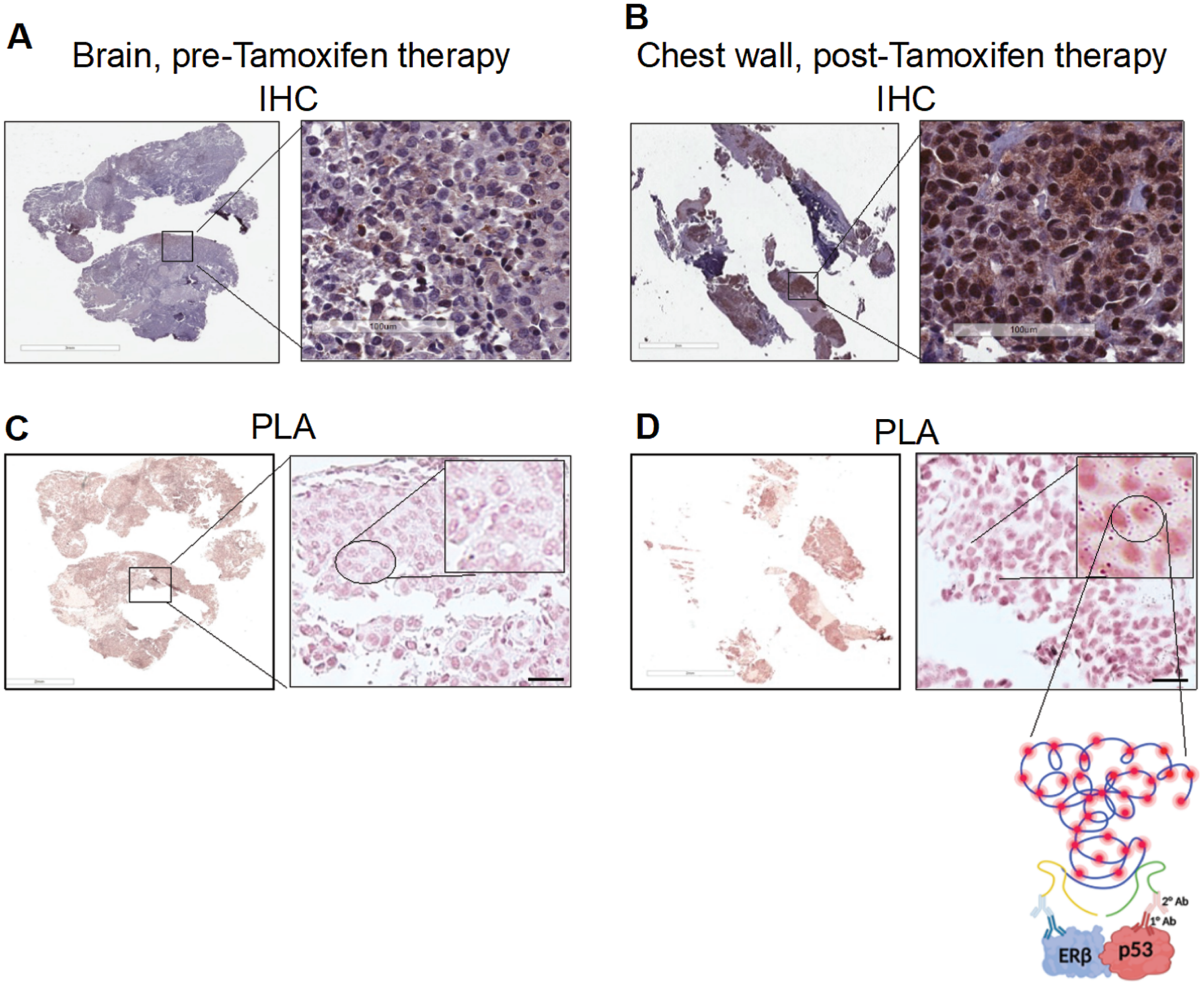
Proximity ligation assay (PLA) assessing estrogen receptor beta (Erβ) (ESR2)-p53 interaction. (**A**): Immunohistochemical analysis (IHC) on brain tumor tissue pre-tamoxifen (12/17/2019), demonstrating ERβ expression. (**B**): IHC of chest wall tumor tissue post-tamoxifen (02/04/2020), demonstrating ERβ expression. (**C**): PLA on brain tumor tissue pre-tamoxifen (12/17/2019), demonstrating lack of ERβ:p53 interaction. (**D**): PLA on chest wall tumor tissue post-tamoxifen (02/04/2020), demonstrating of ERβ:p53 interaction. Inset: Magnified view of interaction dots.

Immunohistochemical (IHC) analysis of ERβ protein expression in the pre-tamoxifen treatment brain tumor specimen showed weak expression of the protein (Fig. 2A) and analysis of ERβ-mutant p53 interaction in the tumor specimen by proximity ligation assay (PLA) demonstrated no interaction between the 2 proteins Fig. 2C). Contrastingly, high levels of ERβ protein expression (Fig. 1B) and increased interaction between ERβ and mutant p53 proteins (Fig. 2D) were observed in chest wall tumor tissue post-tamoxifen therapy.

## Discussion

Here we report an example of efficacy of tamoxifen in metastatic mutant p53 TNBC, demonstrating both clinical activity as well as pharmacodynamic inhibition. These findings add to the evidence suggesting a role of estrogen modulator therapy in enhancing ERβ levels and the ensuing antiproliferative ERβ-mutant p53 interaction.

**Figure 3.**
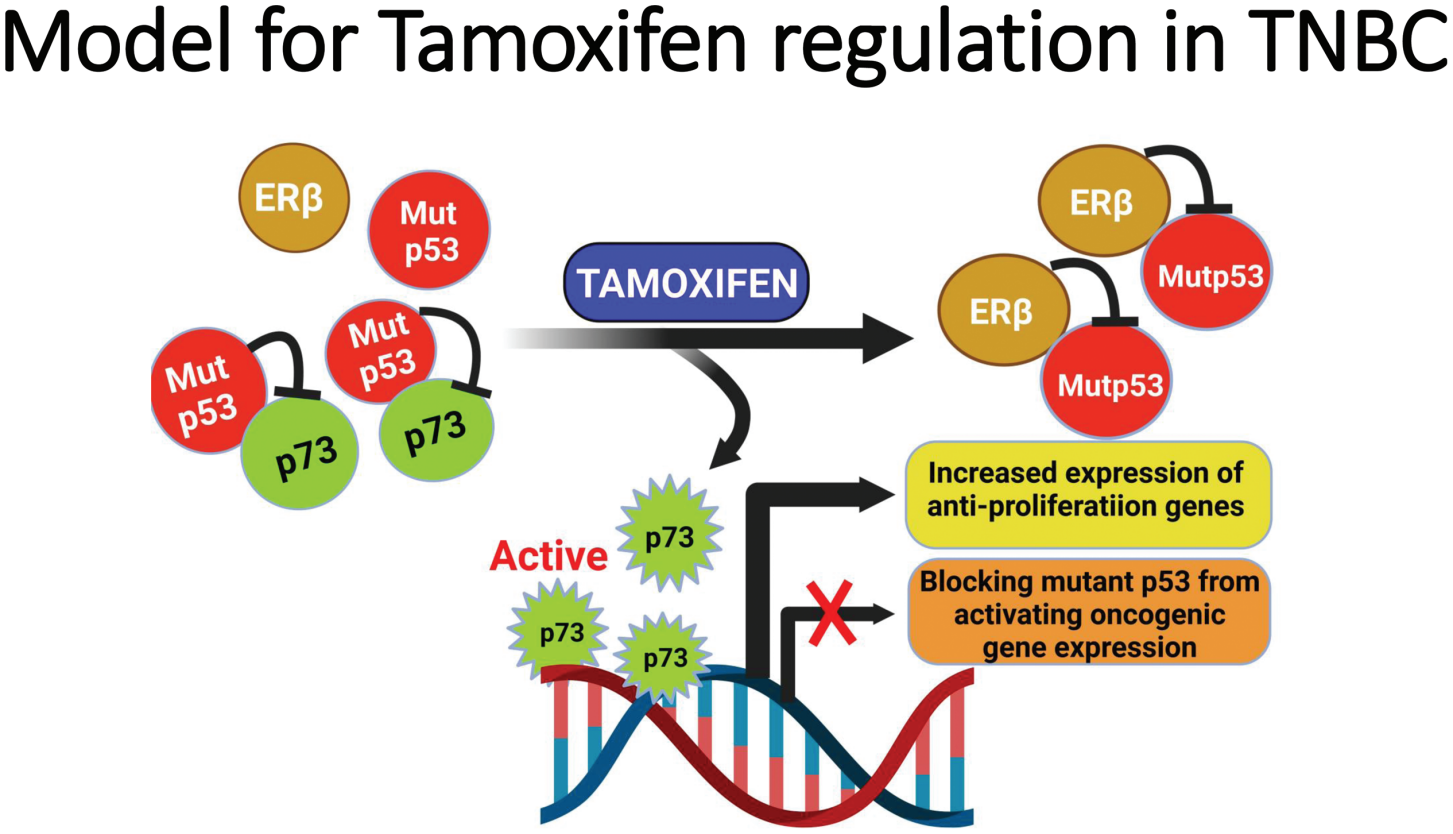
Schematic model depicting how tamoxifen enhances estrogen receptor beta (ERβ) binding and sequestration of mutant p53 away from the mutant p53–p73 complex resulting in reactivation of p73 as a tumor suppressor. Activated p73 mediates up-regulation of anti-proliferation gene expression while blocking mutant p53 from activating oncogenic gene expression. This phenomenon ultimately leads to anti-tumor growth and metastasis response. *This figure was created with BioRender.com*

Tamoxifen is an anti-estrogen that selectively blocks estrogen binding to ERα through competitive inhibition.^25–27^ Though tamoxifen is mainly used in adjuvant therapy of ERα-positive breast cancers, some clinical studies have shown that this drug was effective in some ERα-negative tumors.^28^ ERβ has been shown to be a predictive marker for favorable prognosis in response to adjuvant tamoxifen therapy of ERα-negative tumors including TNBC.^3,29–32^ However, none of these studies have taken into consideration the status of p53 in the tumors. The data from the current clinical study corroborate with our preclinical studies that showed treatment of TNBC cells with tamoxifen increased interaction between ERβ and mutant p53 leading to decreased cellular proliferation.^14^ A diagrammatic model depicting the mechanism underlying the effect of anti-tumor effects of Tamoxifen on TNBC is shown in Fig. 3.

In the case of brain metastases, estrogen has been shown to reduce adaptive immune response as well as phagocytic capacity of microglia via polarization to the M2 phenotype, promoting metastatic growth even in ER-negative disease. Recent mouse models have demonstrated that tamoxifen inhibits M2 polarization and brain tumor progression, suggesting tamoxifen may be an effective treatment for TNBC with brain metastases.^33^

Current therapy for brain metastases in TNBC now includes monoclonal antibodies and antibody–drug conjugates such as trastuzumab-deruxtecan and Sacituzumab govitecan.^34,35^ At the time this patient was undergoing treatment, these options were not standardly available. It has been hypothesized that tamoxifen in combination with monoclonal antibodies for TNBC may be more effective than either treatment in isolation, due to synergistic antiproliferative mechanisms. However, preclinical models examining tamoxifen in combination with TRA-8, a monoclonal antibody targeting death receptor 5, have demonstrated antagonistic effects.^36^ Additional preclinical research on the efficacy of tamoxifen in combination with monoclonal antibodies is required before this regimen is to be considered in clinical practice. At present, we demonstrate that tamoxifen alone has significant antiproliferative effects in ERβ+, p53-mutant TNBC. Until additional research is performed, tamoxifen should be considered as a monotherapy in recurrent TNBC, particularly for patients who do not respond to antibody and/or antibody–drug therapies.

Due to the invasive nature of CNS biopsy, a chest wall sample was obtained for posttreatment evaluation of ERβ expression and interaction with mutant p53. Of note, the exact same point mutation as well as an 18 bp deletion in p53 were present both in the CNS and chest wall tumor tissues indicating that these tumors at different locations did not evolve from different clonal populations. However, because of the different cellular and tissue contexts, tumor heterogeneity between the chest wall and CNS metastases must be considered when drawing comparisons. The identification of *TP53* mutation in the right cerebellar lesion prior to treatment and significant tumor regression after tamoxifen therapy allows for the conclusion of an ERβ-mutant p53-mediated antiproliferative mechanism with reasonable certainty.

While TNBC commonly recurs within 3 years of primary diagnosis,^37,38^ this patient’s recurrence was 14 years after the identification of the second TNBC primary in 2005. It is possible that her profound response to tamoxifen was interrelated to the indolent nature of her disease, in that the antiproliferative effects of ERβ-mutant p53 interactionare more robust in slow-growing tumors. Further research on the use of tamoxifen in more aggressive TNBC is required.

In summary, we demonstrate a potential therapeutic role of tamoxifen in a molecularly defined subset of TNBC. These novel findings require confirmation in a larger study.

## Patient Update

The patient is currently doing well with no evidence of disease progression noted on last set of restaging scans (June 2022).

## Data Availability

The data underlying this article will be shared on reasonable request to the corresponding author.

## Dedication

Dedicated to the oncology research community that continue to strive toward making advances in oncology care and treatment. We especially want to acknowledge the Research Nurses who tirelessly support their patients, families, and research providers. Research Nurses have a unique opportunity to make observations and can concurrently drive the advancement of nursing science through independent research, further contributing to the medical literature.

## Appendix: Materials and Methods

### Immunohistochemistry of ERβ

For antigen retrieval, patient’s tumor tissue slides were either heated in a steamer for 40 minutes in citrate buffer (pH 6.0) for staining. Endogenous peroxidase was quenched with aqueous 3% H_2_O_2_ for 10 minutes. Slides were then loaded on a DAKO autostainer where they were treated with serum-free protein block (DAKO, cat #X0909, CA, USA) for 5 minutes followed by the primary antibody of ERβ (14C8, Gentex, Irvine, CA, USA) incubation for 1 hour. Secondary antibodies, HRP-conjugated anti-mouse (Leica, cat # PV-6114, NJ), were used for 30 minutes. Slides were developed by the 3,3’-diaminobenzidine (DAB) chromagen (DAKO, cat # K3468 CA, USA) for 10 minutes and, finally, counterstained with hematoxylin, dehydrated, cleared, and coverslips were mounted on the slides. After overnight incubation, slides were scanned by Leica Aperio ScanScope XT, and images were captured using Aperio Spectrum Digital Slide System.

### Bright-Field Proximity Ligation Assay (Bright-Field PLA)

FFEP tumor tissues are collected from patients and processed for Bright-Field PLA. Tissues were deparaffinized in xylene for 3 minutes and hydrated through an ethanol gradient (3 minutes in 100%, 3 minutes in 70%). After hydration, tissue was washed twice with tris-buffered saline with Tween 20 (TBST) followed by antigen retrieval using antigen retrieval buffer (EnVision FLEX Target Retrieval Solution, High pH (50x) (Dako Omnis) GV804) in steamer at 100 °C for 1 hour. Tissue slides were cooled at room temperature and further quenched by Duolink hydrogen peroxide (Sigma-DUO82054) for 10 minutes followed by blocking by Duolink blocking solution (Sigma-DUO82007) for 5 minutes. Primary antibodies of ERβ (GeneTex 14C8) and FL393 (SantaCruz Biotechnology, Dallas, TX) were applied on each tissue and incubated further for 1 hour in a humidified chamber. Following the primary antibody incubation, secondary PLA antibodies were applied to the tissue and incubated for 1 hour in a humidified chamber followed by ligation and amplification reactions as described by the manufacturer (Sigma-DUO92012A). After amplification, tissues were incubated with Horseradish peroxidase (HRP)-labeled hybridization probe for 1 hour in a humidity chamber at 37 °C followed by incubation with substrates (A, B, C, and D) as per the manufacturer protocol (Sigma, Cat # DUO92012B). Following 2 washes of distilled water, tissues were then counterstained for 1 minute in Duolink nuclear stain (Sigma, Cat # DUO82059). Tissue sections were dehydrated (2 × 3 minutes in 95% ethanol; 2 × 1 minute in 100% ethanol) and transferred to xylene (3 minutes) and coverslips were mounted using mounting media (Fisher scientific, Cat # 23425401). After overnight incubation, slides were scanned by Leica Aperio ScanScope XT and images were captured using Aperio Spectrum Digital Slide System.
